# Is Palm Kernel Cake a Suitable Alternative Feed Ingredient for Poultry?

**DOI:** 10.3390/ani11020338

**Published:** 2021-01-29

**Authors:** Mohammad Naeem Azizi, Teck Chwen Loh, Hooi Ling Foo, Eric Lim Teik Chung

**Affiliations:** 1 Department of Animal Science, Faculty of Agriculture, Universiti Putra Malaysia, Serdang 43400, Selangor, Malaysia; naimazizi83@gmail.com (M.N.A.); ericlim@upm.edu.my (E.L.T.C.); 2 Department of Pre-Clinic, Faculty of Veterinary Science, Afghanistan National Agricultural Sciences and Technology University, Kandahar 3801, Afghanistan; 3 Institutes of Tropical Agriculture and Food Security, Universiti Putra Malaysia, Serdang 43400, Selangor, Malaysia; 4 Department of Bioprocess Technology, Faculty of Biotechnology and Biomolecular Science, Universiti Putra Malaysia, Serdang 43400, Selangor, Malaysia; hlfoo@upm.edu.my; 5 Institute of Bioscience, Universiti Putra Malaysia, Serdang 43400, Selangor, Malaysia

**Keywords:** broiler, growth performance, nutritional value, poultry, palm kernel cake, solid-state fermentation

## Abstract

**Simple Summary:**

Supply of raw materials such as corn and soybean meal as livestock and poultry feeds may be limited and is a significant concern during the Covid-19 pandemic especially for the countries that depend on importation of raw materials. Consequently, the palm kernel cake has been proposed as an alternative raw material for animal feeds to reduce importation dependency. The chemical composition of palm kernel cake varies depending on the method of oil extraction. The crude fiber content of palm kernel cake is acceptable to most ruminants but is considered high for poultry. Biodegradation of palm kernel cake through solid-state fermentation can improve its nutritional quality, improving broiler health status and growth performance.

**Abstract:**

Palm kernel cake (PKC), a by-product of oil extracted from palm nuts through expeller press or solvent extraction procedures is one of the highest quantities of locally available and potentially inexpensive agricultural product. PKC provides approximately 14–18% of crude protein (CP), 12–20% crude fiber (CF), 3–9% ether extract (EE), and different amounts of various minerals that feasible to be used as a partial substitute of soybean meal (SBM) and corn in poultry nutrition. Poultry’s digestibility is reported to be compromised due to the indigestion of the high fiber content, making PKC potentially low for poultry feeding. Nevertheless, solid-state fermentation (SSF) can be applied to improve the nutritional quality of PKC by improving the CP and reducing CF content. PKC also contains β-mannan polysaccharide, which works as a prebiotic. However, there is a wide variation for the inclusion level of PKC in the broiler diet. These variations may be due to the quality of PKC, its sources, processing methods and value-added treatment. It has been documented that 10–15% of treated PKC could be included in the broiler’s diets. The inclusion levels will not contribute to a negative impact on the growth performances and carcass yield. Furthermore, it will not compromise intestinal microflora, morphology, nutrient digestibility, and immune system. PKC with a proper SSF process (FPKC) can be offered up to 10–15% in the diets without affecting broilers’ production performance.

## 1. Introduction

The livestock and poultry industries are vital to global industries that recorded consistent growth over the last 30 years. The poultry industry has relative advantages of being simpler in management, higher productivity, and faster return on investment than other livestock production [1]. The worldwide consumption of poultry products such as meat plus eggs is currently high and tends to grow continuously compared to other livestock products [2]. Poultry meat is an essential source of animal protein in human diets. Besides being moderately cheap and readily available, its composition makes it a necessary part of healthy and balanced diets [3]. The poultry sector is an integral part of Malaysia’s livestock industry. Hence, Malaysia has been one of the world’s highest poultry meat-consuming countries, with annual consumption of poultry meat reaching up to 50 kg/person [2].

Costs of feeds have always been a significant concern in local poultry production [4]. Poultry production costs have continually increased because of the fluctuation prices of high-quality raw materials such as soybean, corn, and others [5]. Several initiatives have been put in place, such as finding cheaper and locally available materials as partial substitutable protein and energy sources instead of SBM and corn in poultry feed formulations. Various locally available and low-priced feedstuffs have been proposed but have not been satisfactorily acceptable for poultry and consequently used in small proportions [6]. Fluctuations in price and comparatively inadequate SBM supply, particularly during the Covid-19 pandemic, lead to city lockdown, and logistics limitation [7,8]. As a result, the discovery of alternative protein sources for poultry feeding diet has become a particular focus in the current scenario to decrease reliance on SBM as the main ingredient of protein in poultry feeds [9].

Generally, most agricultural industries produce large amounts of residue every year. If waste is not effectively managed, it could contribute to the environment and harm both human and animal health. Waste products from the palm oil extraction industry constitute a huge problem, and any initiative towards economic utilization is a positive step towards solving environmental pollution [10]. Some countries around the world produce plenty of local alternative feedstuffs which are by-products of the agricultural industry. More often, these materials such as wheat bran, rice bran, cottonseed meal, copra meal, PKC, palm kernel meal (PKM) wheat pollard, cocoa pods, oil palm fronds (OPF), palm oil mill effluent (POME), sugarcane bagasse, and cassava waste are widely used as livestock feeds [11,12]. Most of these by-products contain significant amounts of anti-nutritional factors (ANF) which are also considered as non-starch polysaccharides (NSPs) [11]. Locally, large quantities of PKC have been produced from the oil palm industry, and comprehensive studies are needed to assess its potential as alternative protein sources for poultry feeds [2].

Being the primary source of vegetable oil production, the oil palm tree (*Elaeis guineensis*) is one of the most critical trees in many tropical countries such as Brazil, Indonesia, Malaysia, Thailand, Columbia, and Ecuador [13]. PKC is a by-product after oil extraction, which is generally a good source of fat, protein, minerals, and carbohydrate [14]. Many studies have been conducted to assess PKC’s feeding value and determine its effects on feeding diets in broiler chicken’s industry. Malaysia is one of the world’s largest palm oil producers with a large amount of readily available PKC. There is a need to use PKC in the poultry industry as a source of protein and energy [15]. The main objective of the palm industry is to generate palm oil. PKC derives from the nuts of palm fruits is generated as by-products [16].

PKC usage is common in ruminant diets but limited in non-ruminant, especially in poultry diets due to its high fiber content [6]. PKC can be a promising feedstuff for poultry feed because of its moderate CP (16.43%) content and energy [17]. Cellulolytic bacteria can significantly improve the nutrient quality of PKC through SSF [18,19]. Therefore, this review paper will critically shed light on the composition and nutritional value of PKC, SSF in PKC, and the utilization of PKC as a poultry feed and its effects.

## 2. Composition and Nutritional Value of Palm Kernel Cake (PKC)

PKC has been accepted as one of the components in animal feeds. Its nutritional values, attractive prices compared to other meals, and long-term availability make PKC more competitive in the international meal market [20].

There are two methods of palm oil extraction: expeller or screw press, and solvent extraction. PKC is the result of the expeller oil extraction procedure, while the solvent extraction technique yields PKM. Extraction with solvent generally produces less residual oil than the expeller process, whereas crude protein and crude fiber are higher in solvent-extracted PKM [21,22]. Therefore, the nutritional values of PKC and PKM differ depending on their method of extraction [10].

More than 75% of PKC are made from cell-wall components, which made up of 35.2% mannose, 2.6% xylose, 1.1% arabinose, 1.9% galactose, 15.1% lignin, and 5.0% ash [23]. β–mannan is the main component of palm kernel by-products NSPs which is regarded as a prebiotic and is known to enhance birds’ immune system and reduce pathogenic bacteria in the small intestines [10].

The nutritional profile of PKC is shown in Table 1. It has a low nutritional value; however, processing and conversion through SSF could significantly increase its nutritional values and make it useful for poultry [10,22,23,24,25,26].

### 2.1. Protein and Amino Acid Content of PKC

Table 2 illustrates the amino acid contents of PKC. Digestible amino acids are essential for determining acceptable sources of protein and dietary supplements. PKC can be utilized as a source of protein as well as an energy source [6]. The CP values of PKC range between 14 and 21% and this variation in CP can be attributed to the different processing methods [33]. This level is low for starter diets for young chicks, but it is adequate for older birds that require lower protein diets. Furthermore, due to the poor amino acid content, especially essential amino acids such as lysine and methionine, the nutritional value of PKC is considered very low [26,32].

### 2.2. The Crude Fiber Content of PKC

The fiber inclusion response depends on the source and amount of dietary fiber and dietary characteristics, likewise for the bird’s physiological and health status [34]. PKC is considered as a high fiber co-product [26]. The CF content of PKC, ranging from 16–18%, is acceptable to most ruminants, but it may not be suitable if included at the high levels in poultry or pig diets. Insoluble and soluble fibers present in PKC are the main reasons for a lower nutrient digestibility in monogastric animals [35]. However, the CF content of PKC can be significantly reduced through fermentation [28,36].

Cellulose is the most significant structural component of the plant cell wall [37]. Cellulose, hemicellulose, and lignin represent approximately 20–50%, 15–35%, and 10–30% of plant cell walls respectively on a dry weight basis [38]. Different species of cellulolytic bacteria and fungi can hydrolyze lignocellulose in plant cell walls [39], as a significant fraction of lignocellulose is composed of carbohydrates. Hence it can be used as a source of renewable energy [40]. For instance, mannan degrading enzymes can be added in the broiler diet to breakdown the main polysaccharide component. This will directly improve feed digestibility and feed efficiency [10]. Besides, a combination of various fibro-lytic enzymes can also enhance the saccharification of NSPs [41]. A study has observed that the effect of PKM supplemented with enzyme contributed greater nutrient digestibility compared to enzyme-free diet, resulting in a higher body weight gains and better feed conversion ratio (FCR) among birds fed with enzyme supplemented diets (40). Hydrolysis tests showed that the yield of monosaccharides obtained represented nearly 75% of the total polysaccharides content in PKC [23]. Common monosaccharides composition of PKC includes glucose, fructose, and mannose at 154, 218, and 22.1 mg/100 g, respectively [15].

### 2.3. Mineral Contents of PKC

Table 3 demonstrates the mineral content of PKC. The ratio of calcium to phosphorus and sodium to potassium is low in diets based on PKC. It is necessary to supplement those minerals to meet most animals’ nutrient requirements [42]. PKC is also a better source of Ca, Mn, Zn, and Na than groundnut cake, whereas groundnut cake is a good source for K, Mg, Fe, and P [43].

### 2.4. Energy of PKC

The growth rate of broiler chickens requires an energy-intensive diet to sustain their growth. PKC provides 6.5 to 7.5 MJ/kg metabolizable energy for poultry (Table 4). The total carbohydrate content of PKC is 47.71%, which is higher than that of groundnut cake (28.3%) and cocoa cake (42.1%) [6,43]. Mannan is the main component of PKC NSPs. It was found that 78% of NSPs in PKC are mannan with low galactose substitution, 12% cellulose, 3% glucuronoxylan, and 3% arabinoxylan [10]. Because of its useful properties, mannan is a biodegradable and bioactive polysaccharide that has been of interest to different sectors. Mannan can be further categorized into glucomannan, galactomannan, and galactoglucomannan based on the sugar unit types in mannan chains [44]. However, PKC is an excellent source of raw material for mannose and mannan oligosaccharide production [10,45]. 

## 3. Solid-State Fermentation (SSF) of PKC

SSF is a biotechnological process in which microorganisms grow in solid substrates in the absence of free water. The goal of SSF is to place cultured microorganisms in direct contact with the insoluble substrate to obtain the concentration of the maximum nutrients for fermentation from the substrate [47]. SSF appears to be a possible technology for the production of microbial products. It improves the nutritional value of agriculture by-products produced by agricultural industries as a residue [48].

As a result, SSF is used widely because of its economical and practical advantages over submerged fermentation such as using of wide variety raw materials with an extensive variation of substrate composition and size, low energy expenditure, less expensive, lesser fermentation space, easier control of contamination and high reproducibility [48,49].

### 3.1. Biochemical Aspects of SSF

One important application of SSF is the production of various enzymes such as cellulases, hemicellulases, ligninase, protease, lipase, pectinase, phytase, amylase, and xylanase which are essential enzymes required for biotransformation of PKC [19,47,50]. Besides various physicochemical factors, numerous environmental factors, for instance, temperature, moisture, pH, inoculum type, substrate, particle size, agitation and aeration, oxygen, and carbon dioxide could influence the growth and activities of microorganisms in SSF [47].

### 3.2. Modification in Fermented PKC (FPKC) Due to SSF

Dietary fibers are heterogeneous dietary components that are not hydrolyzed by the digestive enzymes of non-ruminant animals [51]. For proper functioning of the digestive organs, poultry requires a low amount of complex fiber in their diet. The SSF of PKC produces a product that contains low hemicellulose and cellulose concentration but high protein content [19]. Microbial fermentation using bacteria or fungi has been documented to improve agricultural by-products’ nutritional values by altering its composition. Findings from numerous studies suggest that both bacterial and fungal fermentation increase the total protein and decrease fiber contents of PKC [10,16,17,26,50,51]. 

Different bacteria, such as *Bacillus amyloliquefaciens*, *Paenibacillus curdlanolyticus*, *P. polymyxa*, *lactobacillus,* and *B. megaterium* able to degrade cellulose, hemicellulose, xylans, and mannans molecules, thus significantly improve the natural quality of PKC [12]. *Lactiplantibacillus plantarum* strains (especially; *L. plantarum* RG11, *L. plantarum* RI11, and *L. plantarum* RG14 (based on their total score of extracellular hydrolytic enzymes activates) can grow on PKC biomass. It performs synergistic secretions of various extracellular proteolytic, cellulolytic, and hemicellulolytic enzymes essential for the effective biodegradation of PKC [19]. The latest findings showed the effects of *L. plantarum* RI11 on different renewable natural polymers, describing the *L. plantarum* RI11 can be a potential candidate as lignocellulosic biomass degrader. It can produce functional extracellular cellulolytic and hemicellulolytic enzymes in rice straw, molasses, PKC, and soybean pulp [52]. 

On the other hand, the fermentation of PKC by *Trichoderma longibrachiatum* significantly increased CP from 18.76 to 32.79% and decreased cellulose levels from 28.31 to 12.11% [53]. Fermentation of PKC by *Aspergillus oryzae* decreased the hemicelluloses levels from 37.03 to 19.01% [54]. Lateef et al. [36] reported that fermentation of agro-waste by-products by fungal strain *Rhizopus stolonifera* LAU 07 under SSF, increased crude protein level from 19.7 to 26.3% and decreased crude fiber level from 22.5 to 12.5%. Yadi et al. [55] reported that fermented substrate by *Trichoderma viride*, containing 80% PKC and 20% rice bran, increased CP from 13.38 to 17.34% and decreased crude fiber from 30.55 to 23.67%. Nevertheless, the primary concern for fungal fermentation is the production of various mycotoxins in the substrate. Deoxynivalenol, nivalenol, zearalenone, fumonisin, vomitoxin, patulin, aflatoxin, and ochratoxin are few examples of mycotoxins which can depress the growth of animals and could be hazardous for both human and animals. The mycotoxin problem can be prevented by substituting fungi with various cellulolytic bacteria in SSF [12,18,19,56]. 

Briefly, microorganisms will utilize agricultural biomass as raw materials for their growth via the fermentation processes [57]. Hence, the desirable effect of microbial activity in fermented feed is caused by its biochemical activity [58]. Those microbial enzymes will break down carbohydrates, lipids, proteins, and other feed components during the fermentation of PKC, which ultimately improves the overall PKC nutritional quality [12].

## 4. Utilization of PKC as Livestock Feed

Palm fibers are safe as they are pure, non-carcinogenic, free of pesticides, and have soft parenchyma cells that can be processed and produced as animal feeds [59]. PKC is one of the highest quantities of locally available and potentially inexpensive feedstuffs in many tropical countries [27] (Table 5).

## 5. Limitation to Using PKC in Non-Ruminant Nutrition

There is a limitation to using PKC in monogastric animal diets because of the high CF, coarse texture, and gritty appearance. Traditionally, PKC has not been used widely in pig and poultry diets. This mainly because of its unpalatability and high fiber content (150 g/kg DM). As a result, this reduces its digestibility for these animals [17]. The CF content of PKC, ranging between 16% and 18%, is considered high for non-ruminants. It may not be suitable if included at high levels in poultry or pig diets [6]. The presence of high content of NSPs in PKC prevents it from being widely used in poultry diets. Thus, SSF is employed to reduce NSPs [17,35]. Furthermore, PKC has different anti-nutritional factors like 0.40% tannic acid, 6.62 mg/g phytin phosphorus, 23.49 mg/g phytic acid, and 5.13 mg/g oxalate which has adverse effects on the nutritional quality of PKC [43].

## 6. PKC in Poultry Nutrition

Malaysia is one of the world’s largest palm oil producers with abundant PKC available throughout the year. There is a need to efficiently utilize this by-product as an alternative feed for the local poultry industry [17]. The importation cost of corn and SBM dramatically influences the price of animal feedstuff in the country, making PKC an alternative feed ingredient. To poultry farmers, the primary factor in utilizing PKC is its relatively low price to be used as one of the ingredients in poultry diets [62]. The feed cost per/kg decreases with increasing levels of PKC [1,34,63,64]. Nonetheless, the challenge of using agro-byproducts as feed ingredients for poultry is the presence of fiber components in these materials. Since poultry has a simple digestive system, the inclusion of PKC in their feeding diet is limited because of the absence of fiber digestive enzyme activities in their gastrointestinal tract (GIT) [51]. Additionally, some essential nutrients such as amino acids and energy content in the PKC may influence the feed cost.

Few researchers have reported variations of optimum inclusion level of PKC in poultry rations. The use of PKC in poultry depends on the type, age, and sex of the chickens, as well as the sources and variations of oil and shell content of the PKC [6,46]. Edwards et al. [62] suggested that PKC in poultry diets should be limited to 20%. The same finding by Anaeto et al. [1] showed that broiler birds could utilize PKC based diet up to 20% without adverse effects on their production performance. Furthermore, Ugwu et al. [65] also recommended that the 20% PKC can effectively replace maize for the finisher phase of broilers resulting in a better performance.

Furthermore, PKC inclusion in broiler chickens’ diets improved the relative weights of immune organs and enhanced humoral immunity [66]. On the contrary, results obtained by Alshelmani et al. [17,35] showed that the inclusion of more than 10% untreated PKC in broiler diet might have adverse effects on birds’ performance. These contradictory results may be contributed to the oil extraction methods from palm fruits, which led to the differences in its composition. The findings obtained by Zanu et al. [60] showed that layers could utilize PKC based diet better (up to 5 and 10% inclusion) without any adverse effects on their production. In contrast, the egg production was adversely affected consequent to 15% PKC supplementation.

## 7. Effects of PKC on Broiler Growth Performance

In broiler chickens, growth performance is the most important economic factor in their production. It was reported that broiler chickens could tolerate up to 40% of PKM inclusion without adverse effects when those diets were formulated based on digestible amino acids and metabolizable energy [10,67]. Furthermore, another study indicated that the inclusion of 8 and 16% PKM increased weight gain compared to 0% PKC diet, whereas weight gain was severely reduced by feeding 24% PKM diets. Meanwhile, 0 and 16% PKM diets had similar feed intake, whereas feed intake of 8% PKM diet was higher among the groups [68]. Results obtained by Alshelmani et al. [17] determined that 15% PKC in broiler feeding diet led to a significant decrease in body weight gain compared to 0 and 5% PKC. The finisher phase of broilers fed with 10 and 15% PKC had lower growth performance than birds fed with 0 and 5% PKC. Bodyweight gains of birds fed with 10 and 15% PKC were significantly lower than birds fed with the same levels of FPKC and control groups. The FCR was higher (2.07 and 2.16 g:g) for 10 and 15% PKC compared with the same levels of FPKC (1.83 and 1.93 g:g) and control groups (1.91 g:g), respectively. While the body weight gain was significantly lower for chickens fed with 10 and 15% PKC compared to the same levels of FPKC and control groups. Moreover, Rahim et al. [69] using diets containing 25% PKC observed the growth performance of broilers fed with untreated and treated PKC groups was significantly (*p* < 0.05) lower than the broilers fed the control (untreated) diet. These discrepancies of PKM and PKC findings may be due to the differences in oil extraction methods, which are solvent and expeller press that led variation in nutrient composition.

Anaeto et al. [1] have reported the effect of feeding broiler during the finisher phase with 0, 10, 20, and 30% PKC. There was a significant difference in weight gain among the birds, with birds fed 20 and 30% PKC diets had higher weight gain, while feed intake and FCR were not significantly different. In contrast to another study, PKC inclusion at 5 and 10% in broilers’ diet did not adversely affect the body weight, daily body weight gains, and feed intake. In contrast, the inclusion of PKC at 15 and 20% significantly reduced birds performances [66]. Zanu et al. [60] referred that PKC inclusion at 15% level in layers’ diets did not affect the feed intake while significantly reduced body weight gain. A study on Muscovy ducks showed that 35% of PKC’s inclusion significantly increased feed intake and reduced FCR [42]. A research conducted by Pushpakumara et al. [70] showed that weight gain of birds fed with diet containing 20% PKC, was significantly lower compared with birds fed with 10% and 15% PKC. The feed intake of birds fed with 15% PKC was significantly higher than birds fed with 0 and 5% PKC. Meanwhile, the FCR of birds fed with 20% PKC was significantly higher than all other treatments. In contrast, the FCR of birds was not significantly affected by the inclusion of PKC at 5%, 10%, and 15%.

As mentioned before, the inclusion of PKC at a higher level in the poultry diet decreases nutrient digestibility due to the higher fiber content of PKC [17,64]. Kalmendal et al. [71] reported that the presence of high levels of fiber in poultry diets negatively affect the surface, width, and height of intestinal villi, and subsequently affecting nutrient utilization negatively. This reduction in nutrient digestibility may be accompanied by an increase in feed intake [42]. The birds’ poor performance was observed to be a result of high concentrations of neutral detergent fiber (NDF), acid detergent fiber (ADF), CF, and NSPs in the mentioned components [17,72]. Additionally, increased fibers in poultry diets may also increase viscosity and passage of ingesta in the small intestines. Therefore, the utilization of nutrients such as CP, amino acids, and energy would be adversely affected [17]. High levels of fiber in broiler feeding diets could also negatively affect intestinal villi morphology [71]. Hence, short villi resulted in an impaired absorption due to loss of intestinal surface area and, consequently, the overall growth performance [2,51]. The increase in feed consumption by broilers feeding on higher PKC levels could be due to energy dilution in PKC that encourages chickens to consume adequate feed to meet their energy requirements [63,73,74].

An optimum level of PKC or PKM in broiler chickens’ diets to enhance growth performance can be achieved through solid-state biodegradation. It has been proposed that the biomass of PKC or PKM can be treated with microbes. These microbes can produce extracellular proteolytic, cellulolytic, and hemicellulolytic enzymes to improve nutritional values [11,12,15,19,32,33,36,75,76,77]. Additionally, as the fermented feeds improved gut health through proper microflora population and balance of metabolism [78], it may increase the utilization of nutrients like CP, amino acids, and energy. Hence, chicken growth performance could be improved [79]. Results of numerous studies [10,11,41] indicated that the body weight and feed intake of birds fed with 15% PKC were significantly lower compared to the same level of PKC + enzyme. It maybe due to the fact that the mannanase, α-galactosidase, cellulase, and various other fibrolytic enzymes in broilers’ diets increase the degradation of diet and eventually increase the growth performance of birds [41,76]. Briefly, the variations in the effects of PKC on broiler growth performance was noted to be due to the different nutrient composition of PKC arising from differences in methods of oil extraction [21,22].

## 8. Effects of PKC on Carcass Yield and Internal Organs

It has been known that the birds’ dietary protein sources can influence the relative weights of breast, drumstick, abdominal fat, as well as their internal organs (liver, heart, spleen, gizzard and bursa). Findings of Okupe et al. [80] showed that breast weight of birds fed 0% PKC diet was significantly higher compared to those fed with 10, 20 and 30% levels of PKC, whereas drumstick weight of birds fed with 30% PKC diets was significantly higher compared to birds fed with 0, 10 and 20% PKC diet. The result obtained by Okupe et al. [80] showed that the abdominal fat weight of birds fed with 0% PKC diet was significantly higher than those fed with different levels of PKC. Chinajariyawong and Muangkeow [72] reported that the abdominal fat of birds fed on diets containing PKM or FPKM was significantly lower than the control. Alshelmani [17] showed that abdominal fat of chickens fed with 10 and 15% PKC included was higher than 5% PKC and FPKC included groups. Dietary composition and lipid metabolism can greatly influence abdominal fat [81]. The significant increase in abdominal fat for birds fed high levels PKC or FPKC attributed to the inclusion of palm oil with higher ratios in their diets than the control or low levels PKC or FPKC treatments [17]. β-mannan is the main component of palm kernel biomass NSPs [10]. It is reported that 0.5 gr/kg and 1 gr/kg mannan oligosaccharide in broilers feeding diet reduced percentage of abdominal fat in the carcass and did not significantly affect dressing percentage and liver, heart, gizzard and bursa weights of birds [82].

On the other hand, PKC at 0, 5, 10, 20, and 30% inclusion levels in broiler diet did not significantly affect birds’ carcass characteristics [66,83]. Numerous other studies [17,55,77] determined that the inclusion of PKC and FPKC at 5, 10, and 15% in broiler diet also did not significantly affect their carcass characteristics. Similar findings were recorded by Bello et al. [63], Mardhati et al. [84], and Pushpakumara et al. [70], that PKC did not significantly affect the dressing percentage of birds. These variations of PKC effects on birds dressing percentage may be due to different ration types, nutrient content, birds breed, environmental conditions, processing, and management conditions.

It was reported [80] that the liver weight of birds fed with 0% PKC diet was significantly higher than those of birds fed with 10% PKC diet, whereas the weight of heart of chickens fed 0% PKC was significantly lower than birds fed with 10, 20, and 30% PKC diet. Besides, the proventriculus weight of birds fed with 30% PKC diet was also significantly higher compared to birds fed with 10% or 20% PKC diets. Pushpakumara et al. [70] determined that liver weights of birds fed with 0, 5, 10, 15, and 20% levels of PKC were not significantly different among birds fed with varying treatment diets. Besides, Soltan [66] showed that gizzard size and relative spleen weight did not significantly increase at 5% inclusion level of PKC but increased substantially with 10, 15 and 20% inclusion levels in broiler diet compared with 0% PKC group. Zanu et al. [60] showed the same findings that the gizzard of laying chickens increased with 15% inclusion level of PKC. Okeudo et al. [83] reported that gizzard weight was significantly higher in birds fed with 10, 20, and 30% PKC diet compared with the 0% PKC group. The increase in gizzard size could be due to the high fiber content of PKC [85]. An increase in spleen’s relative weight following the feeding of fungal on fermented PKC or PKM could be due to the influence of fungal toxin during SSF [72]. Commonly, the suggested inclusion levels of PKC in broiler diets do not have adverse effects on overall carcass traits.

## 9. Effects of PKC on Gut Morphology and Gut Microflora

### 9.1. Gut Morphology

The intestinal crypt is the dilation of the epithelium around the villi. The base of the crypt is continuously dividing to maintain the structure of the villi. Thus an increase in depth of the crypt creates more developed villi [2]. The height of villi and surface area is important to determine nutrient absorption potential [86]. The density and size of small intestinal villi and micro-villi are directly related to the birds’ ability to absorb the nutrient [87,88]. Diet has an important influence on gut health, including effects on the proliferation of pathogenic bacteria, and it can provide either beneficial or harmful effects [51]. High fiber levels in poultry feeding diets negatively affect the surface, width, and intestinal villi height [5,71]. A fermented feed with low pH, high amount of lactic acid and acetic acid, and an increased number of lactic acid bacteria (LAB) can effectively improve gut health through intestinal microflora balance and development of intestine [89]. 

Alshelmani et al. [35] reported no significant difference in birds’ morphology of small intestine among the different diets (5, 10, and 15% PKC and FPKC) groups. Utilization of FPKC until at level 9% with no significant effects on height and density of villi in all parts of the small intestine, whereas at 18% level, only jejunum villi were significantly lower [5]. Results obtained by Yaophakdee et al. [90] showed that chickens fed PKM at 15% in diet did not affect ileum morphology. Meanwhile, Zulkifli et al. [30] showed that the 25% inclusion of PKM significantly increased the villi height and width. Findings by Sabour et al. [87] indicated that fibrous supplementation increased villus height of broilers chickens. Hence the improvements of gut morphology may be due to the high fiber content of PKC [30,87]. Further investigations are still needed on this topic. 

The chickens’ response with dietary fibers is based on the source of fiber content, diet characteristics, and the bird’s physiologic and health status [34] and duration of the dietary fiber in the diet, animal species, and age of the animal [51]. The performance and gut health of broiler chickens could be improved due to improved nutrient digestibility when SSF was used to reduce the anti-nutritional factors in plant protein sources and increase the bio-availability of nutrients and inhibit pathogenic bacteria in the gut [88]. 

### 9.2. Microflora Count

Gut microflora benefits the host by helping in digestion, absorption, and storage of nutrients. Furthermore, gut microflora control and improve epithelial immune responses and function [2]. The inclusion of palm kernel expeller in chickens’ diet improved non-pathogenic bacteria count in the intestines [10]. For instance, the inclusion of 15% FPKC in broiler feeding diet significantly increased the LAB counts compared to the negative control and different PKC groups’ levels. Simultaneously, no significant difference was observed between the dietary treatments in *Enterobacteriaceae* (ENT) counts [35]. Zulkifli et al. [30] showed that broiler chickens fed with 25% PKM significantly increased counts of *Lactobacillus* sp. and *Streptococcus* sp. in both caecum and ileum. Loh et al. [91] showed that the addition of 6 and 9% fermented products in laying hens feeding diets reduced the ENT population in feces and increased the fecal LAB population.

Dietary treatments have shown to influence the composition of gut microbiota. LAB is the most common bacteria used as probiotics and inhibits the intestinal ENT population [2]. Zulkifli et al. [30] reported that feeding a higher fiber diet may increase gut microflora population. Moreover, fermented products increase intestinal LAB, decrease intestinal pH, and increase lactic acid concentration. It is well established that the dominance of beneficial bacteria in the host’s gut can improve nutrient intake and nutrient absorption [58]. 

## 10. Effects of PKC on Nutrient Digestibility

Generally, poultry feed formulation is based on digestibility and absorption in the diet. It is known that most dietary fibers in PKC are in the form of mannan, which is not hydrolyzed by digestive enzymes of those non-ruminant animals. Therefore, nutrient digestibility decreases with increasing PKC in birds’ feeding diet [10,41,85,92]. It was described that 10 and 15% PKC in broiler diet in both starter and finisher phases significantly decreased digestibility of DM, CP, EE, and nitrogen-free extract (NFE) compared to the negative control. However, there was no significant difference observed in DM, CP, and EE digestibility for 10 and 15% FPKC. Additionally, no significant difference was observed in ash’s digestibility for 10 and 15% FPKC than the negative control. In contrast, ash’s digestibility was lower for 15% PKC than the negative control group [35]. Aya et al. [64] reported that CP, ash, and NFE digestibility values in the control group (0% PKM) were significantly higher than other PKM diets with or without enzyme supplementation. Another study conducted by Fadil et al. [42] determined that the digestibility of DM, gross energy, and CP for 35% PKC included diet in ducks was more impoverished than 0 and 15% PKC diet. 

Dietary protein sources are known to affect broiler nutrient digestibility [93]. It was claimed that CP, CF, and EE’s digestibility decreased with PKC inclusion in broiler diet [80]. The decrease in nutrient digestibility of chickens fed with PKC could be attributed mainly to the lack of any mannan-degrading enzymes in poultry’s digestive system [10]. Moreover, indigestible fiber molecules could increase the passage rate of ingesta and could decrease nutrient absorption. Consequently, high levels of dietary fiber led to a reduction in the digestibility of energy, starch, protein, and lipids in monogastric [51]. Furthermore, insoluble materials in diet could increase the viscosity of intestinal digesta, leading to reduced digestibility and absorption of nutrients [94]. Moreover, Hakim et al. [95], show that bacterial fermentation, enzymatic fermentation and thermal extrusion have the potential to improve the apparent metabolizable energy (AME) of PKC. Both bacterial and enzymatic fermentation enhanced the CP digestibility. However, Lawal et al. [33] reported that feeding treated PKC with enzymes contributed to better nutrient digestibility of broiler chickens.

## 11. Effects of PKC on the Immune System

Mannan oligosaccharide content of PKC plays various biological functions, particularly in minimizing gut pathogens and enhancing poultry’s immune responses [96]. It was reported that mannose and manno-oligosaccharides could act as prebiotics by improving the chicken immune system, reducing the gut’s harmful bacteria and increasing non-pathogenic bacteria population [10]. The result obtained by Shashidhara and Devegowda [97] showed that antibody responses against infectious bursal disease virus (IBDV) were higher in birds fed with manno-oligosaccharides (MOS) supplemented diet. Moreover, the maternal antibody titers in chicks were also significantly influenced by MOS supplementation. Soltan [66] showed that the inclusion of 18% PKC in broiler feeding diet significantly improved relative spleen weight, whereas 5% of PKC non-significantly increased relative spleen weight. Nonetheless, the different inclusion levels of PKC showed no significant improvement in the relative weights of the bursa and thymus gland compared to control. However, feed supplementation of mannanase for broiler chickens improves gut morphology and plasma immunological status [84]. 

Supplementing OligoPKE at 5 and 1% in broiler diet had no effect on plasma immunoglobulin M (IgM) of birds. However, feeding with OligoPKE supplemented diets had higher immunoglobulin A (IgA) concentrations than control [98]. 

Van der Wielen et al. [99] showed that dietary fibers were fermented in the caecum of birds by faecal microbes which produced end products such as volatile fatty acids (VFAs), an essential compound in decreasing of *Salmonella* spp and other pathogenic bacteria. MOS is the main component of PKC and is considered to contain prebiotic properties that can reduce pathogenic bacteria and improve birds’ immunity [10,96,100]. FPKC in the broiler feeding diet may increase intestinal LAB and lactic acid concentration [58]; hence, the interactions between the LAB and the host immune system have been suggested to lead to some immunomodulatory activities [101]. Organic acids, mainly lactic acid in fermented feeds, may increase beneficial bacteria leading to higher production of short-chain fatty acids and ultimately acidify and lower the pH throughout the GIT [89]. Additionally, organic acid can decrease pathogenic bacteria directly through penetration into the bacterial cell wall and produce H^+^ ions that interfere with bacteria’s enzyme activities or indirectly by changing the intestines’ pH. Thus, a cell must spend more energy to maintain internal pH, and this energy cannot be used for other metabolic processes. It may result in less number of pathogenic bacteria [91,102]. Moreover, lowering of pH improved organic acids’ antimicrobial activity against pathogens [103].

## 12. Conclusions

Conclusively, PKC is acceptable to be included in ruminants’ diet, but may not be suitable to be included at high levels in poultry diets owing to the high CF content. In regular farm practice, not more than 6-8% PKC level is included in broiler chickens’ dietary without affecting growth performance, carcass yield, intestinal microflora and morphology, nutrient digestibility, and chicken’s immune system. However, this agriculture biomass could be treated by various microorganisms through SSF, increasing the inclusion level (10–15% FPKC) in the diets without affecting broilers’ production performance. SSF of PKC help to decrease CF levels by degrading the complex carbohydrate fractions. Furthermore, microbial activities in SSF contribute to increasing levels of CP, amino acids, and energy in FPKC. Additionally, the inclusion of biodegraded PKC in broilers feeding diets could ultimately improve gut health, increase nutrient digestibility, and improve chickens’ overall growth performance.

## Figures and Tables

**Table 1:** Chemical composition of palm kernel cake (PKC) (%).

Nutrient	PKC ^1a^	PKC ^1b^	PKC ^1c^	PKM ^2a^	PKM ^2b^	PKM ^2c^	FPKM ^3^	FPKC ^4^	FPKC ^5^	FPKC ^6^	FPKC ^7^
Dry matter	88.57	91.42	90.87	96.3	91.80	91.75	91.83	88.9	92.62	92.40	89.85
Crude protein	16.86	16.43	16.23	16.6	20.04	16.60	23.42	20.7	16.80	16.68	17.11
Crude fiber	15.12	-	-	14.6	15.47	12.29	12.44	11.3	-	-	14.59
Ether extract	6.82	-	4.12	8.3	8.63	7.59	3.89	4.07	-	-	5.15
Ash	6.58	4.47	5.10	4.3	7.56	3.88	8.33	18.9	4.67	4.80	5.40
Gross energy (Mcal/kg)	-	-	4153	4872	-	-	-		-	-	-
ME (Kcal/kg)	-	-	-	-	2792.1	2423	2655.9	2282.7	-	-	-
Nitrogen free extract (NPE)	54.62	-	-	-	-	51.39	-	45.0	-	-	57.75
Neutral detergent fiber (NDF)	-	82.29	61.54	-	-	-	-	39.8	71.70	73.54	-
Acid detergent fiber (ADF)	-	51.48	36.14	-	-	-	-	17.5	47.27	47.45	-
Hemicellulose	-	30.81	25.40	-	-	-	-	22.2	24.43	26.42	-
Cellulose	-	35.55	-	-	-	-	-	8.16	31/85	31.41	-

^1a^ Untreated palm kernel cake [27]. ^1b^ Untreated palm kernel cake [28]. ^1c^ Untreated palm kernel cake [29]. ^2a^ Untreated palm kernel meal [30]. ^2b^ Untreated palm kernel meal [25]. ^2c^ Untreated palm kernel meal [31]. ^3^ Ensiled PKM; PKM was ground, sprinkled with water until wet (not dripping) then ensiled for 7 days [25]. ^4^ Degraded PKC; PKC was sprayed by extracts from *Aspergillus niger* and bags sealed for 7 days [32]. ^5^ Fermented PKC by *Paenibacillus polymyxa* ATTCC 842, for 9 days incubation period [28]. ^6^ Fermented PKC by *Paenibacillus curdlanolyticus* DSMZ 10248, for 9 days incubation period [28]. ^7^ Fermented PKC by *Trichoderma koningii* for 21 days [27].

**Table 2:** Amino acid contents of palm kernel cake (PKC) (dry matter basis).

Nutrient%	PKC ^1a^	PKC ^1b^	FPKC ^2^	FPKC ^3^
Indispensable amino acids:				
Lysine	0.37	0.38	0.41	0.38
Leucine	0.89	0.88	0.94	0.95
Isoleucine	0.50	0.46	0.59	0.53
Valine	0.69	0.65	0.78	0.72
Phenylalanine	0.57	0.61	0.66	0.63
Threonine	0.41	0.51	0.51	0.46
Histidine	0.23	0.16	0.29	0.24
Methionine	0.22	0.30	0.27	0.26
Arginine	1.60	2.01	1.76	1.69
Glycine	0.60	0.71	0.78	0.71
Dispensable amino acids:				
Aspartic acid	1.12	1.32	1.27	1.23
Glutamic acid	2.48	3.34	2.80	2.76
Proline	0.44	0.60	0.59	0.52
Serine	0.56	0.99	0.69	0.66
Tyrosine	0.25	0.35	0.24	0.24
Cysteine	0.20	0.12	0.22	0.21
Alanine	0.62	0.98	0.70	0.71

^1a^ Untreated PKC [28]. ^1b^ Untreated PKC [29]. ^2^ Fermented PKC by *Paenibacillus polymyxa* ATTCC 842, for 9-day incubation period [28]. ^3^ Fermented PKC by *Paenibacillus curdlanolyticus* DSMZ 10248, for 9-day incubation period [28].

**Table 3:** Mineral contents of PKC.

Content	References	
	[42]	[43]
Calcium (%)	0.27	0.25
Phosphorus (%)	0.46	0.33
Magnesium (%)	0.11	0.14
Sodium (%)	-	0.17
Potassium (%)	-	0.16
Copper (ppm)	25.52	-
Zinc (ppm)	53.91	21.40
Manganese (ppm)	259.00	1.30

ppm: part per million.

**Table 4:** Metabolizable energy of PKC.

Metabolizable Energy	(MJ/kg)	(Kcal/g)
Ruminants	10.5–11.5	0.0025–0.0027
Poultry	6.5–7.5	0.0015–0.0017
Swine	10.0–10.5	0.0023–0.0025

Source: [46].

**Table 5:** The recommended inclusion levels of PKC in livestock feed.

Livestock	Recommended Level (%)		References
	PKC	FPKC ^1^	
Poultry—broiler	Up to 10	Up to 15	[17,35]
Poultry—layer	5–10	-	[60]
Swine	15–25	-	[61]
Freshwater fish	10–20	-	[61]

^1^ Fermentation of PKC by *Paenibacillus polymyxa* ATTCC 842, for 9-day incubation period.
